# Reply to: Concerns regarding proposed groundwater Earth system boundary

**DOI:** 10.1038/s41586-024-08083-8

**Published:** 2024-11-20

**Authors:** S. E. Bunn, B. Stewart-Koster, C. Ndehedehe, C. Gordon, J. Rockström, J. Gupta, D. Qin, S. J. Lade

**Affiliations:** 1 Australian Rivers Institute, Griffith University, Brisbane, Queensland Australia; 2 Institute for Environment and Sanitation Studies, University of Ghana, Legon, Ghana; 3 Potsdam Institute for Climate Impact Research, Member of the Leibniz Association, Potsdam, Germany; 4 Institute of Environmental Science and Geography, University of Potsdam, Potsdam, Germany; 5 Amsterdam Institute for Social Science Research, University of Amsterdam, Amsterdam, The Netherlands; 6 IHE Delft Institute for Water Education, Delft, The Netherlands; 7 State Key Laboratory of Cryospheric Science, Northwest Institute of Eco-Environment and Resources, Chinese Academy of Sciences, Lanzhou, China; 8 China Meteorological Administration, Beijing, China; 9 University of Chinese Academy of Sciences, Beijing, China; 10 Stockholm Resilience Centre, Stockholm University, Stockholm, Sweden; 11 Fenner School of Environment & Society, Australian National University, Canberra, Australia

replying to: M. O. Cuthbert et al. *Nature* 10.1038/s41586-024-08082-9 (2024).

Groundwater is widely used for domestic and agricultural purposes, but is subject to increasing risks from overexploitation. Responding to this global significance, we recently defined a safe and just Earth system boundary (ESB) for groundwater^1^ and, in the absence of a consistent data source on baseline aquifer volumes, used derived estimates of groundwater storage (GWS) from the Gravity Recovery and Climate Experiment (GRACE) satellite mission data to assess the state of the Earth system with respect to the ESB for groundwater. In the accompanying Comment^2^, Cuthbert et al. agree that our effort to define a groundwater ESB is timely and important, but they assert that our approach is “flawed, unsafe and unjust”. Their concerns reflect misunderstandings of the definition and purpose of the boundary, a misunderstanding of the ‘safe’ and ‘just’ concepts^3^ that underpin our work, a lack of confidence in the use of GRACE data to calculate changes in GWS, and the possibility of confusion related to the use of some terms.

Our definition of the subglobal ESB is independent of the data source used to assess the current Earth system state relative to the boundary. We defined the safe and just ESB as met where net annual drawdown from all sources (which includes natural discharge to surface waters and anthropogenic abstraction) does not exceed net average annual recharge to prevent a long-term downwards trend in groundwater levels^1^. Our motivation was to choose ESBs that maintain the stability and resilience of the Earth system (for groundwater, to sustain groundwater-dependent ecosystems) and to avoid harm to humans (for groundwater, for example, from seawater intrusion or land subsidence)^1^. We reported on the current GWS trends of the global land surface area using the GRACE data (Table 1 in ref. ^1^), in which groundwater storage change provides an indicator of changes in the groundwater level, but it is important not to conflate the assessment of the current state of global GWS with the definition of the ESB. The spatial definition of the ESB addresses concerns about the limitation of the solely volumetric approach to previous derivations of the planetary boundary for water^4^.

Cuthbert et al. suggest that previous groundwater pumping that may have created a new dynamic equilibrium and previously devastated ecosystems could be considered safe under the proposed ESB. We explicitly acknowledged (page 47 of Supplementary Information in ref. ^1^) that a limitation of using averages defined during the current climate is that “it sets present/recent levels of recharge as the baseline, which fails to account for harm that is already caused to present generations”. Indeed, we state (Table 1 of in ref. ^1^) that the safe and just ESB must also “ensure recovery” and, as such, we disagree that an accurate interpretation and application of the safe and just ESB would allow historical over-extraction to be considered ‘safe’. We recognize that this is not systematically explored in the paper and we welcome suggestions on how to better assess this.

It is true that a decrease in the GWS of an aquifer is likely when its subglobal ESB is not met. However, this does not imply that no new abstraction is possible under safe conditions, as suggested, because the ESB is defined (page 107 in ref. ^1^) as “Annual drawdown does not exceed average annual recharge”. Some new abstraction may be possible within the ESB in years with above average recharge. The ESB does take into account natural variability and will change as average annual recharge responds to climate and other factors.

Cuthbert et al. note that GW depth can be lowered and attain a new equilibrium in response to sustained pumping, but this cannot be considered safe under our definition because of the potential impact on groundwater-dependent ecosystems (for example, ref. ^5^) and base flows to rivers. We also agree that a downwards trend in annual recharge (which is the actual basis for the ESB and not GWS) may occur for reasons that have nothing to do with pumping. Indeed, our subsequent work^6^ shows that some regional declines in groundwater recharge are associated with declining trends in annual rainfall.

The critique that this is a flaw of the groundwater ESB indicates a misunderstanding that the ESBs were never intended to provide a sustainability assessment. The ESBs, across all domains considered in ref. ^1^, delimit states of the Earth system that ensure planetary stability and minimize significant harm to people from Earth system change. Although the ESBs can help inform target-setting, the groundwater ESB is not intended to provide a target for sustainable groundwater pumping. We used the GRACE data to determine the broad trends in GWS decline; however, the groundwater extraction that may safely occur within this boundary “should be defined based on local-scale monitoring”^1^ including assessments of capture across a suitable reference period and groundwater pumping hydraulics as noted by Cuthbert et al.

Cuthbert et al. contend that the proposed groundwater ESB is also not ‘just’. They first argue that our approach is unjust because “some groundwater storage depletion is inherently necessary to extract groundwater”. This position, however, ignores that ‘just ESBs’ were defined as those that avoid significant harm to people from Earth system change (no significant harm). Our work emphasized that avoiding significant harm is a necessary but far from sufficient condition for justice, which involves many other elements such as minimum access and allocation of resources, risks and responsibilities^3^. Just minimum access to resources and whether sufficient access to resources is possible within safe and just (no significant harm) boundaries are explored in detail elsewhere^6,7^.

The second argument of Cuthbert et al. regarding justice is that already over-abstracted aquifers may be within the ESB. We have addressed this above and acknowledge in the paper that adhering to the safe and just boundaries would considerably restrict current use and require policies to ensure distributive justice. A similar argument is true for climate. The planet has already exceeded the safe and just ESB of 1 °C, but further emissions are necessary for just access to energy under current modes of energy production. As with groundwater, further boundary transgressions will occur if we are to provide just access to all people, without radical and systemic transformations^6^.

The authors state that our estimate of the current global state of the groundwater ESB (that 53% of global land areas currently satisfies the ESB, calculated by quantifying the total area showing groundwater storage decline) should not be considered a “meaningful statement”. They cite a recent study that used GRACE data to assess persistent vertical displacement of land associated with GWS decline^8^ and note that the spatial patterns in that paper differ from those we have mapped in other work^6^. This is not entirely surprising because the two papers have measured related but different processes over slightly different timescales. The broad similarities of surface area with groundwater decline, however, does suggest some confidence in the use of GRACE data, albeit with associated uncertainty.

Cuthbert et al. raise concerns about our use of GRACE data to assess trends in GWS, questioning its observational range and post-processing requirements, including the need to deduct other terrestrial water stores, and our recognition of the uncertainties in the approach. Our approach to quantifying groundwater is based on a consistent, recognized methodology^9,10^ that allows the large-scale assessment of diverse subsurface water storage systems with data provided by NASA/Center for Space Research at 0.25° resolution. We undertook the following steps:

1. As explained in the Supplementary Information of ref. ^1^ (pages 19–20), we followed methodologies to subtract all other water storage components via the Global Land Data Assimilation (GLDAS) National Oceanic and Atmospheric Administration Land Surface Model.
2. We then used trend analyses at all pixels across the globe, following methodologies previously used for country^11^ and global^12^ trend analyses of 0.25° GRACE data. Recent methodological developments include approaches to resolving GRACE solutions that show very strong correlations between GRACE solutions at different scales, including 0.25° (ref. ^13^).
3. Our final step was to quantify the fraction of global land area where the trend in GWS is declining. Proceeding to an aggregated global-scale result is consistent with the advice quoted by Cuthbert et al. to not use GRACE data to analyse an isolated single pixel or a basin smaller than 200,000 km^2^. We recognize that we did not, however, assess the uncertainty introduced by aggregating in this manner.

We recognize the uncertainties in the auxiliary global model data products (for example, GLDAS) usually used in processing groundwater from GRACE, and the challenges associated with spatial resolution, among other issues^14^. These global models generally tend to underestimate water storage changes compared with GRACE^15^ but the GLDAS product has been widely preferred to help quantify groundwater change from GRACE^9,10,14,16^. Furthermore, a review of GRACE analyses validated against local groundwater monitoring found errors to be within 2–13% of the trend signal in large basins around the world (>140,000 km^2^)^17^. Although we discussed these issues in the Supplementary Information of ref. ^1^, we agree that a formal uncertainty analysis should be a high priority for future work.

Cuthbert et al. further argue that nomenclature is a problem and could lead to confusion. We have used nomenclature from the remote-sensing hydrology community where total storage has been used to define groundwater availability (for example, ref. ^14^). Our use of recharge as net annual aquifer water gains (or groundwater availability) and drawdown as net annual aquifer losses from both human abstraction and natural discharge are also consistent with this literature (for example, refs. ^14,18^). We are aware that these definitions differ from those frequently used in hydrological modelling communities and hope that this exchange helps reduce future confusion when these communities interact. We also acknowledge that these definitions should provide an underestimate of groundwater recharge, as stated by Cuthbert et al., and note that our GRACE-derived recharge estimate (in millimetres per year) is indeed lower than other global estimates cited in Supplementary Table 4 in ref. ^1^, but within the range of other global volumetric estimates.

Our paper aimed to be a “transparent proposal for further debate”. We thank Cuthbert et al. for engaging with our work and fully expect and hope that others will take up the challenge in subsequent peer-review publications. The safe and just ESBs for blue water have been developed to protect the Earth system and the ecosystem services that aquatic ecosystems provide and minimize significant harm to humans from changes to blue water flows. The critique of Cuthbert et al. adds further support to the importance of advancing safe and just planetary and Earth system boundaries for freshwater, but we strongly refute their assertion that the current groundwater ESB is “flawed, unsafe and unjust”.

## Data Availability

No new data were created or analysed for this paper.
